# Providing a Smart Healthy Diet for the Low-Income Population: Qualitative Study on the Usage and Perception of a Designed Cooking App

**DOI:** 10.2196/11176

**Published:** 2018-11-23

**Authors:** Faustine Régnier, Manon Dugré, Nicolas Darcel, Camille Adamiec

**Affiliations:** 1 Institut National de la Recherche Agronomique Alimentation et Sciences Sociales Unité de Recherche 1303, University of Paris Saclay Ivry-sur-Seine cedex France; 2 AgroParisTech UMR 914 Physiology of Nutrition and Ingestive Behavior Paris France

**Keywords:** health, cooking, mobile phone, low-income population, social networks, utilization

## Abstract

**Background:**

Health behaviors among low-income groups have become a major issue in the context of increasing social inequalities. The low-income population is less likely to be receptive to nutritional recommendations, but providing cooking advice could be more effective. In this domain, taking advantage of digital devices can be a bonus with its own challenges.

**Objective:**

The aim of this study was to develop and deploy NutCracker, a social network–based cooking app for low-income population, including cooking tips and nutritional advices, aiming at creating small online communities. We further determined the usefulness, perceptions, barriers, and motivators to use NutCracker.

**Methods:**

The smartphone app, designed jointly with beneficiaries of the social emergency services, was implemented in a disadvantaged neighborhood of Magny, (Paris region, France). Once the app became available, 28 subjects, living in the neighborhood, tested the app for a 6-month period. Logs to the app and usages were collected by the software. In total, 12 in-depth, semistructured interviews were conducted among the users and the social workers to analyze their uses and perceptions of the app relative to their interest in cooking, cooking skills, socioeconomic constraints, and social integration. These interviews were compared with 21 supplementary interviews conducted among low-income individuals in the general population.

**Results:**

NutCracker was developed as a social network–based app, and it includes cooking tips, nutritional advice, and Web-based quizzes. We identified barriers to uses (especially technical barriers, lack of knowledge in the field of new technologies and written comprehension, and search for real contacts) and motivators (in particular, good social integration, previous use of social networks, and help of children as intermediaries). Cooking skills were both a barrier and a lever.

**Conclusions:**

Targeting the low-income groups through a cooking app to promote healthier behaviors offers many advantages but has not been fully explored. However, the barriers in low-income milieu remain high, especially among the less socially integrated strata. Lessons from this intervention allow us to identify barriers and possible levers to improve nutrition promotion and awareness in deprived areas, especially in the time of social crisis.

## Introduction

### Background

Current health promotion campaigns that aim at motivating individuals toward healthier eating habits are inefficient among low-income populations. This situation is particularly concerning in the current context where social inequalities in health are very marked and are rising in western countries [1]. Health behaviors among the lower strata appear, more than ever, as a major issue. In the context of a deep economic crisis, leading to increasing health and food consumption inequalities [2], low-income populations appear to face more food insecurity than before [3]. They eat less recommended food products, especially fruits and vegetables [4].

Moreover, public health campaigns, developed by the French National Nutrition and Health Program, focusing on the effect of a proper diet on health, were not able to reach people in low-income categories. Although the disadvantaged populations are well aware of the guidelines for healthy eating [2,5], they do not implement them because of their social lifestyle, tastes, and preferences.

Consequently, we observe an unequal prevalence of obesity [2] and diabetes [6], much more frequently in low-income population, leading to the need to focus on these disadvantaged social categories.

There is need of more efficient strategies to enhance nutritional awareness and promote healthy behaviors in disadvantaged populations through novel interventions specifically targeting them. Hence, the use of interventions that include a cooking component seem to be an effective way to enhance the adoption of better eating behaviors [7], especially among low-income individuals [8].

### Objectives

Digital technologies could offer a good platform to help build and disseminate appropriate individualized recommendations targeting special social groups. As described by the European Commission’s Green Paper on mobile health (mHealth) [9], mHealth solutions appear as a solid basis for people’s empowerment. However, not many culinary apps with health promotion prospects have been developed for mobile devices. NutCracker—continued in the FacilEat4All project—is an interventional research project based on the development of a cooking app to promote healthy nutrition in low-income groups and on its evaluation. This app was jointly designed with the final beneficiaries.

The project took place in disadvantaged neighborhoods of Magny (Yvelines, Paris Region, France). This city had a total population of 32,639 inhabitants in 2015 [10], mainly of a low social level, with 24.6% of its population living below the poverty threshold (national average 14.1%) [11]. The rate of unemployment is high (19.4% vs national average 10.4% [11]). Participants in this study were volunteers recruited via social care services and local nongovernmental organizations (NGOs).

In this study, we aimed to (1) present the coconstruction of NutCracker, specifically designed for low-income people, promoting a healthy diet; (2) describe the results of its usage and determine the barriers and levers of this process; and (3) discuss the main lessons that can be drawn from this study.

## Methods

### App Design: Recruitment and Coconstruction

Our study was composed of 2 parts—an app design and an app evaluation—and was based on 2 subsamples.

We worked with the disadvantaged households of Magny, Paris region, France to design a mobile app for healthy cooking and nutrition. The app was jointly designed with its future beneficiaries in a 2-step process. First, questionnaires were distributed among 46 participants with the help of the local social workers. The questionnaire focused on the socioeconomic status of the respondents, the foods commonly consumed, food supply, expectations and general perception toward food, importance of eating, importance granted to nutritional issues, level of culinary skills, and the availability of cooking equipment. Second, a collective discussion with 8 participants was organized to discuss about a new mobile phone app providing healthy and affordable cooking advices and recipes to determine the participants’ expectations toward such a device. The objective of this discussion was to launch a collective dynamic as well as to reach a consensus on the overall design of the mobile app and provide general recommendations on its features. We used the *persona* method [12] to remove social inhibition stemming from individuals own situations [13]. During the collective discussion, which was audio-recorded and transcribed, we followed a discussion guide in which a series of 4 fictional characters (established on the categories derived from the previous questionnaires), representing an individual that might use the mobile app, was introduced. Participants were then asked to give their opinion on the features of the app that would best suit this *persona*’s needs and expectations. Following a suggestion by 1 of the participants, others were asked to add their thoughts and voice their opinion about this suggestion and to go into as much detail as possible. The outcomes of these sessions were used as the outline for the app development (see Multimedia Appendix 1).

When this app was released, it was tested by 28 volunteers (8 participants from the collective discussion plus 20 supplementary volunteers) from the same neighborhood and recruited in collaboration with beneficiaries of 3 social emergency services in Magny.

### App Evaluation: Recruitment and Semistructured Interviews

Data on individual app usage (logs and visited features) were collected, and a qualitative study was performed to assess this app.

**Figure 1:**
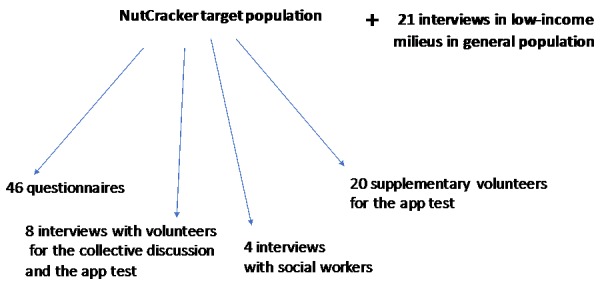
Subsamples of the study.

We conducted 33 semistructured interviews using the 32-item Consolidated Criteria for Reporting Qualitative Research checklist [14]. To evaluate the usage and perceptions of the participants on the NutCracker app, a field study, including semistructured, face-to-face interviews, was conducted on individual project participants (12 in total, comprising 8 users and 4 social workers who were involved in the project; Figure 1). Our interview guide contained a series of open-ended questions about their use of the app, their favorite features, the barriers encountered and, more generally, their use of digital devices and the Web with regard to cooking and in other areas, that is, social networking, gaming, Web-based purchases, and performing administrative tasks. All participants were from the municipality of Magny.

Along with this sample, and to compare with digital practices in low-income population, 21 interviews were conducted in the Paris region, among individuals of the lower strata (Figure 1). We investigated these individuals’ exiting attitudes toward and acceptance of the digital world and their use of new technologies in all their forms. This additional sample allowed us to determine whether the field results for NutCracker revealed specificities linked to the app or if they reflected a larger, shared trends related to digital cooking tools in deprived areas.

These interviews, conducted by the 2 project sociologists, lasted on average of 1 hour, were face-to-face interviews, were recorded (except for 1 participant who refused), transcribed in their entirety by a team of transcription consultants, and anonymized. Analyses of the data were double-checked by the 2 sociologists, and then discussed and validated with the team involved in the project (ie, the 4 researchers who were directly involved and the scientific committee of 5 experts). We developed a content analysis, and the 3 main themes investigated using interview guide were as follows: (1) uses; (2) barriers and levers in using NutCracker; and (3) digital devices in the field of cooking, diet, and other related areas. Among the themes, subthemes were identified in line with the themes in the interview guide, and new themes derived from the data collected were also included.

### Samples Included in the Study

Our study was based on 2 subsamples (Figure 1).

In the NutCracker sample (Table 1), participants were all women, which further shows that in low-income families, women are often responsible for domestic tasks related to food [15]. These were women from underprivileged backgrounds, who were foreign housewives. Their living conditions were unstable and highly dependent on social services. Due to unemployment, the social integration of these women was based entirely on the social group they belong to and on family integration [16]. In comparison, the general population sample contained individuals from low-income categories who were better off socially, either because of having a job (they were employed either as blue collar or employee) or because they lived in more socially diverse municipalities than Magny. Participants from both samples were aged between 28 and 58 years, with a median age of 46 years.

### Ethical Consideration

For the collective discussion, participants were informed about the purpose of the discussion before the interview. For the semistructured interviews, the goals of this study were explained to the interviewees, and individual consent was obtained for the recording. Personal data from the interviews, including the name of the place the project took place in, were anonymized.

**Table 1:** Sociodemographic characteristics of the participants.

Sociodemographic variables		NutCracker target population (n=8), n (%)	NutCracker social workers (n=4), n (%)	Low-income milieus in the general population (n=21), n (%)	Total
**Sex**					
	Male	0 (0)	2 (50)	3 (14)	5
	Female	8 (100)	2 (50)	18 (86)	28
**Age (in years)**					
	19-29	0 (0)	1 (25)	7 (33)	8
	30-39	2 (25)	2 (50)	5 (24)	9
	40-49	5 (63)	1 (25)	5 (24)	11
	50+	1 (13)	0 (0)	4 (19)	5
**Occupation**					
	Intermediate professions	0 (0)	4 (100)	0 (0)	4
	Employee	2 (25)	N/A^a^	12 (57)	14
	Manual workers	0 (0)	N/A	4 (19)	4
	Unemployed	6 (75)	N/A	2 (10)	8
	Students	0 (0)	N/A	3 (14)	3
	Total	8 (100)	4 (100)	21 (100)	33

^a^N/A: not applicable.

## Results

### Joint Design of the App

The results from analyzing the 46 questionnaires emphasized the importance of food abundance and variety, the desire to please children with food, and the value put on national brands as markers of food quality. Although generic nutrition recommendations are very well known, the importance of nutritional issues, such as the criteria for food choices, appeared to be less important for the low-income population than for people with higher social ranking. Respondents declared the frequent use of mobile technologies, with 46 owning a smartphone (ie, 72%, whereas the national average was 58% in 2015 [17]). We noted a substantial use of social networks (ie, Snapchat, WhatsApp, Facebook, Twitter, and Instagram). When questioned about their expectations from a *nutrition program*, respondents pointed to the need for tips on cheap and healthy foods. Indeed, in the questionnaire, 18 out of 46 responded that they had insufficient fund to eat well, and 26 responded that they would eat better quality foods if they had more money (maybe as an echo of the several public health campaigns launched by the French National Nutrition and Health Program). Using leftovers appeared in the top 5 responses to the question on what they would like to find in a mobile nutrition app. Respondents also indicated the need for cooking tips (1 of the top 5 responses to the question on their expectations from a mobile nutrition app). The keys for better understanding of nutritional information and food labeling were also part of the expectations, with 9 of the 46 respondents claiming they had insufficient knowledge of nutrition. During the collective discussion, most of the participants (6/8) hinted on the importance of providing the price or low budget labeling choices for the suggested recipes as well as tips to cook with leftover food. They also asked for practical solutions to meet the dietetic advices: theoretical knowledge in health and nutrition had to be, for the respondents, translated into cooking tips and daily assistance. The mobile app created, based on this groundwork, consisted of 4 features: (1) a collaborative Web-based recipe book, which included recipes with inexpensive and good nutritional quality foods; (2) a series of simple nutritional information and cooking tips; (3) a collection of nutritional quizzes; and (4) a social network component allowing users to share, like, and comment on recipes, or share their achievements in the quizzes. The app was also designed to allow the collection of logs and usages from individual users. Data on the number and dates of logs to the app per user; number of viewed recipes per user; quizzes taken and percentage of correct responses; and recipes entered, shared, and liked were collected.

Once the app was created, it was released and made available for download to all study participants. A total of 28 households who met the selection criteria mentioned above were selected to use the mobile app over a period of 6 months (from May 2016 to October 2016).

### Limited Uses of NutCracker and New Technologies

The postintervention field research highlighted a very limited use of NutCracker among its intended target population, namely, the women (Table 2). Of the 28 women who were presented with the app, only 7 downloaded it, 4 used it, and only in 2 was, thus, a regular occurrence. Only 1 participant used NutCracker actively, published recipes (9 recipes shared), and answered quizzes (she answered more than 200 questions).

By contrast, the app was much more successful among the general population, where it was shared through a network of interconnections. Between May 2016 and October 2016, 378 connections were made, 600 recipes were viewed, 18 recipes were shared via the app, and responses to 1200 quiz question were supplied by the users.

Generally, participants from Magny had more limited access to the internet and to new technologies than participants from the low-income milieus in the general population (Table 2). Participants in the general population used the internet to access cooking websites and to perform Web-based trade, games, or administrative tasks more frequently. Diet or physical self-tracking devices were not used by the target population.

### Barriers to the Use of NutCracker and New Technologies

On the basis of the inductive thematic analysis methodology described in the study by Peng et al [18], several types of reasons for not using NutCracker were identified (Table 3).

#### Technical Barriers

The most frequent barrier cited during the interviews was the technical instability of the prototype NutCracker leading to recurrent interruptions during the sessions. Another factor was the necessity to provide user name and password at every session as well as not being able to quickly reset the password.

**Table 2:** Uses of NutCracker and new technologies by the NutCracker sample and the low-income milieu population.

Uses	Verbatim	NutCracker target population (n=8), n (%)	Low-income milieus in the general population (n=21), n (%)
NutCracker app	“[NutCracker], I don’t have it on my phone anymore, I don’t know why. I had downloaded it and it was really interesting.”	4 (50)	Not applicable
Cooking apps or websites	“Marmiton website...it’s complete, and it works really well.”	5 (63)	14 (67)
Web-based trade	“I sell things on eBay.”	0 (0)	5 (24)
Web-based games	“I play poker on the Internet.”	0 (0)	3 (14)
Administrative Web-based tasks	“My bank accounts, I have everything, the RATP, I have WhatsApp, etc. and I even have Carrefour, Wish, I have my accounts, I have Mappy to get around, I have music stuff.”	4 (50)	11 (52)
Diet or physical activity self-tracking	“I use the app MyFitnessPal.”	0 (0)	1 (5)

**Table 3:** Barriers to using NutCracker and the new technologies by the Nutcracker sample and the low-income milieu population.

Barriers	Verbatim	NutCracker target population (n=8), n (%)	Low-income milieus in the general population (n=21), n (%)
Technical barriers	“I don’t think my telephone is good enough...I will change it, but I don’t know when [...] It’s another expense.”	3 (75)^a^	10 (48)
Unfamiliarity with new technologies	“Administration services computerize, but people are lost. Not everyone has an email address.”	4 (50)	5 (24)
Comprehension and written barriers	“We can speak French well, we know how to write, but there are things that we have trouble deciphering.”	4 (50)	2 (10)
Time constraints	“As I work mornings and evenings, I don’t have a lot of time. [...] I don’t have the time.”	3 (38)	3 (14)
Competition from television	“I watch (the Samira channel) all the time - as soon as my children leave, I watch it.”	3 (38)	2 (10)
Search for real contacts	“Cooking workshops give you the opportunity to get out from of home and meet other people.”	8 (100)	1 (5)
Live social network	“So, do you like it (what I published)? I’m the only one who publish, it’s a shame.”	1 (13)	Not applicable
Fear of being stalked on the internet	“There are always dangers. And I’m scared of...[...] I don’t like it, it’s scary. And...well [...] it can cause damage.”	4 (50)	4 (19)
Interest for cooking and cooking skills	“I have everything in my head.”	6 (75)	13 (62)

^a^We only considered the 4 participants who used the app.

These technical barriers, although unique to NutCracker, reflect an exacerbated trend of limited use or underperformance, if it was not a defective equipment in the low-income milieus. A total of 9 individuals mentioned these issues, which were essentially caused by the insufficient availability of memory or an overtaxing app, because most individuals preferred to keep what space they had for personal photos or videos:

I don’t have many apps on my telephone, I have to buy a card, I don’t have enough memory. All this stuff takes space!

General population, 43 years, secretary, 2 children

#### Lack of Knowledge: New Technologies, French Language, and Written Comprehension

A lack of knowledge on new technologies was also an obstacle. In the least well-integrated fringes of low-income categories (unemployed women and education level lower than a bachelor degree), everyday internet tasks are neglected, unless they attend specific training programs in social centers where they learn how to create and use an email account and a password on a computer before moving to the mobile phone usage.

All women in the NutCracker intervention group were foreigners, and some of them only had a basic command of French or were not comfortable with the written French (4 of 8). These 2 barriers explained both the low rate of usage of NutCracker and the overall use of digital technologies:

I am taking French courses [...] because there are some words that I don’t understand. I can read, but I don’t understand. [...] I have some difficulty.

NutCracker, 44 years, housewife, 4 children

This lack of knowledge regarding the new technologies did not appear as a real barrier to our sample in the general population.

#### Time Constraints

The project participants who did not download NutCracker were characterized by a few financial constraints as they were either unemployed or earned a very low salary. They also had time constraints. For the unemployed women, domestic tasks, most notably those with large families, were cited as barriers to using NutCracker. Eveline and Hawa were employed, but they had long commutes to work, and their workdays as housekeepers were split in 2 (early mornings and evenings).

#### Competition From Television

In Magny, 3 of the women interviewed expressed a preference for passive media (television), which allowed them to have their hands free, unlike a smartphone, tablet, or computer. It gave them the opportunity to do household chores, with the television playing in the background.

Regarding cooking more specifically, and for the most underprivileged participants in our study, television was the main source of culinary expertise: television channels, such as the Algerian channel Samira, which is entirely devoted to cooking and gastronomy, were greatly valued.

Among the participants from the general population, the television rarely prevented them from using digital devices.

#### Search for Social Contact in Real Life

The NutCracker app was aimed to connect women from underprivileged areas to Web-based micronetwork; however, the interviews with these women showed that they preferred to socialize *in the real world.* Given their extreme social isolation, these women sought social contact outside of the domestic realm [17]. Workshops in social centers where they were introduced to the app represented a place of freedom for them. This attachment to *real* encounters was associated with low use of digital communities.

This refusal of being part of virtual socializing was observed in only 1 participant from the low-income milieus sample; she was better positioned socially, but her real-life socializing was affected by a break-up. Following her divorce, Elga was seeking stronger social ties:

There are already so many things that we can buy on the internet, if we add food to that, we will never leave the house. I need to get out of the house.

General population, 47 years, employed, 2 children

#### Large Number of Members of a Living Social Network

The small number of members of a living social network prevented the creation of an active and thriving social network. Individuals, such as Martine (NutCracker, 39 years, housewife, 3 children), who were the most enthusiastic, found themselves to be quite alone in their investment in NutCracker, which led them to stop using the app. For example, in her interview, Martine said that her enthusiasm waned once she realized that she was one of the few active people in the network and that no one was responding to her posts.

#### Fear of the Internet and of Participation in Social Networks

The NutCracker app depended on women publishing their recipes; however, women expressed reluctance to publishing recipes and to sharing their own experience. Furthermore, putting one’s own recipes on the Web implies exposing oneself, as food is closely connected to identity, whether individual or social [19]. It is quite likely that for many of these women, publishing a recipe was seen as exposing an intimate realm, that of their home cooking or their cultural origins, which they perceived as being devalued in a migratory context.

This also explains why publishing recipes as part of a group in a workshop was much easier than doing so individually. Instead of involving risky personal exposure, the collective posting was perceived as developing a collective identity:

...putting up their recipes, they were happy, it was gratifying.

NutCracker, social worker

In the general population, participants more rarely mentioned the fear of a digital tracking (4 of 21), and in these cases, individuals expressed a fear of being overwhelmed by the internet and becoming addicted to digital tools. Furthermore, participants from a better social position presented limiting the use of digital tools as a choice; thus, the refusal of digital technologies was chosen, and not endured.

### Levers for Using NutCracker and New Technologies

The results below show some levers for using NutCracker and new technologies (Table 4).

#### Social Status and Familiarity With New Technologies

As expected, the participants who were more familiar with new technologies were the ones who most easily took to the NutCracker app, which was related to the social position of the individuals. They had a slightly higher social status (most notably through their husbands’ professions in the case of the 2 most active users); a higher level of education (high school qualification), which in itself requires a certain familiarity with digital and information technology devices; a functional equipment (a powerful smartphone or a home personal computer); and a keen interest in cooking. This outcome was confirmed by our general population sample, where participants had higher social positions (in terms of occupation or level of education), and almost all participants used the internet and new technologies.

#### Former Uses of Social Networks and Leadership

The participants who were most active in NutCracker were also those who were already familiar with social network apps most used in low-income areas (Table 4). This Web-based sociability was bolstered by already well-developed real-life sociability. Martine represents a case study in this area. Martine’s social insertion proved to be successful, as it was strongly linked to local social bonds, notably within the NGOs, alongside a high level of Web-based sociability (on Facebook, Twitter, Snapchat, Pinterest, and YouTube).

Furthermore, Martine characterized herself through her position as an intermediary, which drove her to invest herself in the digital culinary realm, whether on NutCracker or, previously, other sites or social networks (especially Marmiton or her personal Facebook page where she published recipes). Originally from Senegal, Martine arrived in France a few days after she was born:

I’m familiar with French culture, as well as my homeland’s culture, so I quite enjoy a kind of mixing of both cultures and all, yes, that’s me.

This distinguished her from the other participants who had arrived in France much later in life, and often as adults. This sense of double belonging, associated with pride in her 2 cultures, led her to see cooking as a way of exchanging and discovering new cultures. This positive self-image also helped her feel more comfortable publishing her personal recipes on the Web.

#### Children as Intermediaries

The interview study highlighted the role of children as essential intermediaries for digital technologies as they were regarded as the experts in the field. All the more, as there is a tendency for the underprivileged to please their kids with the symbols of consumer society [5], which now includes mobile devices, quite often, the women’s mobile phone was not capable of connecting to the internet, whereas the children have smartphones. Kelthoum is proud of her children’s expertise:

I know how to use it, but there are a lot of things we don’t know [...] but young people, they know everything.

NutCracker, 44 years, housewife, 2 children

Eveline’s case was interesting as well:

It’s my daughter who knows, because she’s the one who installed my [Viber] app (...) She knows how to do everything on the internet. 

Among the low-income milieus in general, children appear as mediators, and they may help in case of technical difficulties, but their role was not as essential as in the deprived households, and they were rarely mentioned in the discourses we collected (once only).

**Table 4:** Levers for using NutCracker and new technologies by the NutCracker sample and the low-income milieu population.

Levers	Verbatim	NutCracker target population (n=8), n (%)	Low-income milieus in the general population (n=21), n (%)
Social integration	“I’m an outgoing person...I’m too outgoing [...] I’m organizing a pastry competition (at school) for the mums.”	2 (25)	17 (81)
Knowledge of new technologies	“Compared to before, we had no Internet...we just had a blank shit [...] Now, everything is	4 (50)	18 (86)
Familiarity with other social networks	“I already had a Facebook page precisely on cooking, I was used to publish recipes every week.”	6 (75)	10 (48)
Children acting as intermediaries	“It’s the kids who know about that.”	2 (25)	2 (10)
Digital and modernity	“I’m a geek of my smartphone.”	1 (13)	5 (24)
Videos on internet	“But there’s everything on YouTube [...] You just type “chicken” or “Tajine” and it gives you [...] there are tons of different videos.”	3 (38)	7 (33)
Interest for cooking and cooking skills	“Maybe I’ll find some ideas (on the app) in order to make my children eat vegetables.”	6 (75)	15 (71)

#### Added Value of Videos on the Internet

Those participants who were the least comfortable with the French language preferred using visual material on the internet or in apps to increase their culinary expertise (3 of 8 in the NutCracker group); watching cooking videos on YouTube was preferred over using websites or apps that were predominantly text-based. Thus, Fatima (Magny), who had difficulty with the French language, used YouTube videos—the links were sent to her by her friends via WhatsApp:

I start the video, I put it on full screen (...) it’s faster, it’s easier.

NutCracker, 44 years, housewife, 4 children

In addition, when she looked something up on the internet, she used the *microphone* function on Google so that she did not have to write anything.

This approach was also a trend we observed in low-income milieus in the general population (eg, Rabia, 38 years, employee: “I watch videos (on YouTube) for recipes”) that was exacerbated in Magny.

#### Cooking Skills: Both a Barrier and a Lever

Cooking skills appear as both a barrier and a lever. The 2 women who actually used NutCracker had a keen interest in cooking—for Martine, the appeal was more on the *cooking* side and, for Kelthoum, who was mostly looking for fast and easy ways to get her children to eat vegetables, it was more on the *health* side. She explained why she really got into the project:

Maybe I’ll find a way to get my children to eat my food, vegetables included. [...] There are a lot of moms who have trouble getting their kids to eat vegetables.

NutCracker, 43 years, housewife, 3 children

On the other end of the spectrum, great culinary traditions based on oral transmission rendered the use of the internet unnecessary and unattractive when it came to cooking. Eveline echoed this when explaining why she did not need the internet to nourish her culinary inspiration:

It’s all in my head.

NutCracker, 44 years, employed

Exactly the same words (“it’s all in my head”) were expressed in the general population by Savina (45 years, old manual worker).

Furthermore, in this specific social milieu, cooking was not necessarily connected to the digital domain, as emphasized by a social worker in Magny:

...making the connection with the app is very, very difficult.

Cooking is the realm of the tangible and the emotional, which is disconnected from the digital world. It is difficult to determine, at this stage of our investigation, whether this separation is specific to people from underprivileged groups or not.

Finally, and somewhat unexpectedly, those who were completely uninterested in cooking were the ones who demonstrated the most interest in the app; they figured that the digital tool could provide them with ideas to turn what they saw as a tedious task into something more fun. For Clarisse, cooking was like:

a chore; when we get home from work, we’re tired.

Therefore, regarding an app:

Yes, why not? It could provide some ideas.

General population, 54 years, employed, 2 children)

## Discussion

### Principal Findings

NutCracker, developed as a social network-based app, includes cooking tips, nutritional advices, and Web-based quizzes. We identified barriers to its uses (including technical barriers, lack of knowledge in the field of new technologies and written comprehension, and search for real contacts) and motivators (in particular, good social integration, previous use of social networks, and help of children as intermediaries). Cooking skills were both a barrier and a lever. Although the designers of the project had great expectations and foresaw a high membership rate, the real results may appear disappointing. Several explanations can shed light on this discrepancy. To begin with, a social desirability bias was linked to the group’s situation in the collective discussion, where the participants found it difficult to express reluctance toward a project concerning a pleasant and well-liked area such as cooking and the symbols of modernity, that is, digital tools. Furthermore, the participation of these women in this project revealed a desire to move upwards socially in very underprivileged areas by gaining access to information deemed good and desirable (eating healthy, at a low cost) as these recommendations by the National Program for Nutrition and Health have been widely promoted since 2001 [2]. For these women, culinary innovation and having access to affordable recipes containing ingredients promoted by public health campaigns (fruits and vegetables) to prepare for their children are ways to project themselves as being *good cooks* and *good mothers* and to conform to the values and practices of those who are more well off. Finally, the prospect of using a socially valued tool gives these women the feeling of participating in, and benefitting from, the consumer society in the same way as individuals from more privileged backgrounds.

The reality of the field study itself also revealed some barriers. First, those related to the slowness or capacity issues of the devices, especially because smartphones were often the only internet access for the households in our study. Our work corroborates previous studies, which noted that the speed the apps use was critical to the satisfaction of users [20]. Studies also show that although digital tools are relatively common among the French population, the distribution of smartphones is much more unequal than that of mobile phones [21], which our study corroborates.

We have also added some new elements to the digital divide such as the difficulties experienced by our participants, which reflect the working class’s unfamiliarity with new technologies. Our results demonstrated that the lowest percentile of the low-income population, which is the least socially integrated, are at greater risk of digital inequalities, which is in line with previous reports [17,22]. Conversely, a good social integration promotes the use of cooking apps and the knowledge of social norms affecting food choices [23] and uses of new technologies [22].

Our study confirms a gap in the use of digital self-tracking tools. Although individuals from well-off backgrounds choose to use these tools [19], individuals from underprivileged backgrounds are compelled to use them. The low usage rate of NutCracker by its target population was mainly because of constraints in time and equipment, whereas individuals, who were more socially integrated, including those from underprivileged areas, exercised their choice and decision-making power in using the digital world.

This research also confirms the ability of this app to act as a culinary lever to promote healthier eating habits [7,24] through the interest of people in using digital tools and not through providing health information. Cooking apps are among the tools often used in underprivileged areas, whereas self-tracking devices, based on a dietary and quantified approach, are not familiar.

However, the connection between cooking and the digital world is very weak and should be explored further in future. Our results highlight the widespread reliance of the target population on videos, which allow them to overcome the written-language barrier and to rematerialize cooking through direct access to tips and tricks, emphasizing the significance of visual material. The use of videos to develop cooking skills [25] is currently being tested in the FacilEat4All project, which is expanding on the NutCracker project.

Finally, we have demonstrated the difficulty experienced by women of very underprivileged backgrounds to integrate and participate in online social networks, barring women who were already well-integrated in their real world and were active on social media. Social inequalities were increased by gender inequalities. Our results corroborate studies performed on digital technologies that highlight the complementarity and overlapping between real-world and Web-based sociability [26,27]. To compensate for the difficulty in creating an autonomous ex nihilo social network, our team suggests the use of existing social networks through a private Facebook group to foster a sense of community spirit more quickly. Recent work has shown the circulation of a prevention program based on social networks [28]. Finally, the NutCracker experience shows the relevance of individuals-as-intermediaries; their investment in digital tools makes them opinion leaders, and their importance has recently been emphasized in the field of electronic health [29,30]. We consider the position of cultural intermediary to be crucial to this investment, which made Martine a *champion* of the app [31].

### Limitations

The app was disseminated among a small sample of participants: this prevented the creation of an active social network. Our current research project FacilEat4All—a continuation of NutCracker—is based on a broader community (approximately 100 volunteers). Another limitation of the NutCracker project is that the choice of the participants, which was limited to individuals from underprivileged backgrounds, led to the selection of people with heavy combination of constraints (budget, social integration, and language). The FacilEat4All project now includes people from modest categories with less difficulties (white or blue collars, in socially more diverse areas).

Finally, the method of face-to-face interview was particularly interesting for the postintervention study to have access to individual opinions on the app. On the contrary, collective discussions appeared as useful tool to launch a collective dynamic for the project, albeit the group’s discussion led to a general consensus regarding the expectations concerning the app itself.

In the most underprivileged milieus, it was particularly difficult to motivate individuals to test the app over a long term (3 months) and to participate in the social network. One conclusion of this limitation is that in those underprivileged milieus, digital devices are more easily accepted if used as collective tools, in workshops for example.

### Conclusions

The NutCracker and FacilEat4all projects bring new elements to a theme that had not been studied closely until now. They promote healthy eating through culinary levers and digital tools. The NutCracker app and the study of the use and perception of digital *diet* tools (nutrition or cooking) by people with underprivileged backgrounds have highlighted the numerous barriers in using cooking apps for people from modest backgrounds. In addition to technical barriers, a lack of skills related to new technologies, a reluctance toward written material, and a combination of time and financial constraints also restricted the use of the app. What stopped the participants from inserting themselves into the online social micronetwork were difficulties regarding self-expression on the Web and a need to integrate socially in real life.

Our study also shed light on the levers we rely on, such as prior experience and use of social networks, which led certain participants to become leaders. We also observed the importance of children as intermediaries of new technologies. Finally, our research has demonstrated the importance of culinary levers in the development of digital tools for people in the low-income categories. Further interventions should assess the advantages of a cooking-based communication platform to promote healthier behaviors.

## Appendix

Multimedia Appendix 1 Screenshots of a website; SPSS files containing original data.

## Abbreviations

mHealth: mobile health
NGO: nongovernmental organization
