# Supplementary material for: Providing a Smart Healthy Diet for the Low-Income Population: Qualitative Study on the Usage and Perception of a Designed Cooking App
Source: JMIR Mhealth Uhealth. 2018 Nov 23;6(11):e11176. doi: 10.2196/11176 (PMC6286424; doi:10.2196/11176)

Supplementary data: description of the mobile applications.

The mobile app consisted of a personal dashboard and four features: (1) a collaborative online recipe browser including recipes with inexpensive foods and good nutritional quality with the ability to search by name(s) or by ingredient(s); (2) a series of simple nutritional information and cooking tips; (3) a collection of nutrition quizzes; and (4) a social network component allowing users to share, like, and comment on recipes or share their achievements in the quizzes.

1. Personal dashboard

To access the application the user had to create a personal account first.

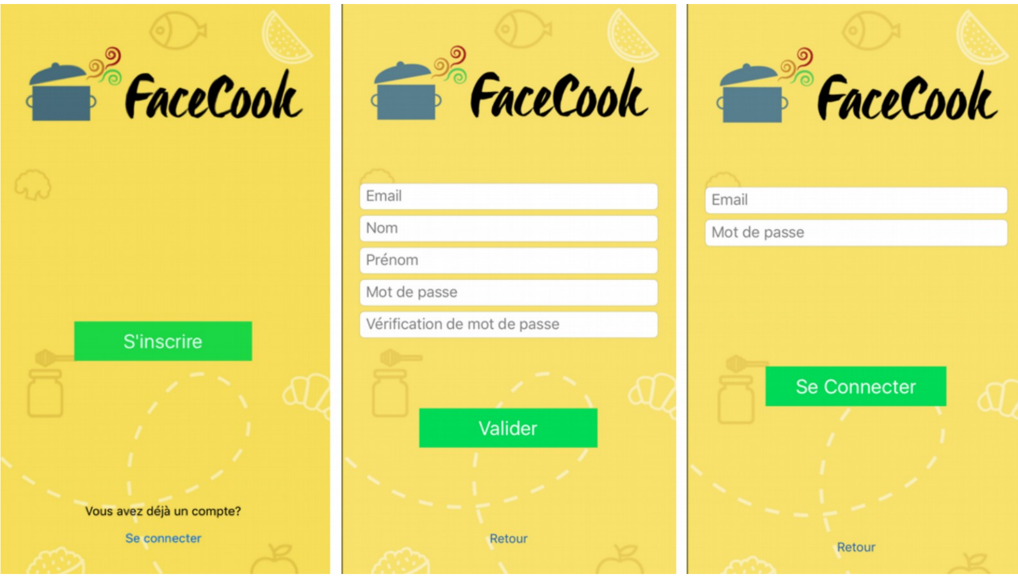

The users then provided information on their preferences or specific diets.

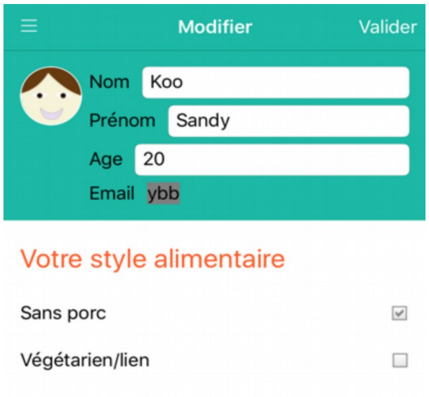

At any time, users could visit their personal dashboard, which showed the number of recipes entered and liked and the level of experience of the user.

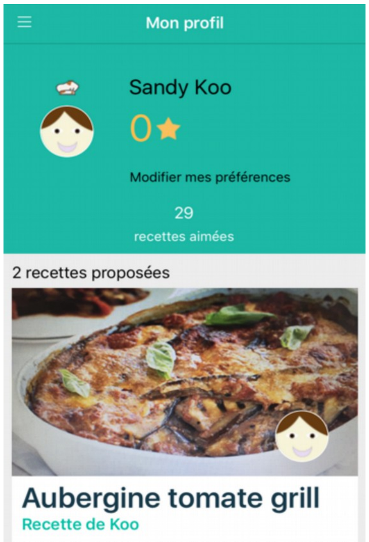

The more recipes the user accesses the more experienced the user became. This “level of experience” was indicated by a chef’s hat ranging from very simple to very elaborate.

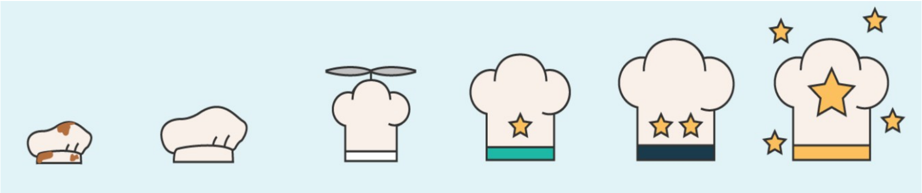

A side sliding tab was available to simplify browsing throughout the application, by providing direct access to the recipe browser, quizzes or nutritional tips and advices.

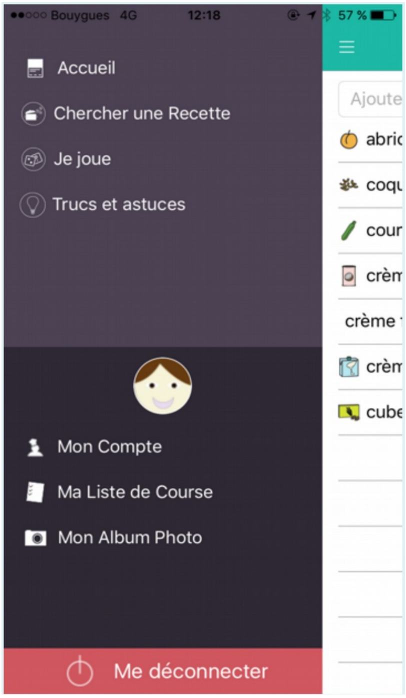

2. Collaborative on-line recipe book

There were four ways to search for a recipe:

- by name

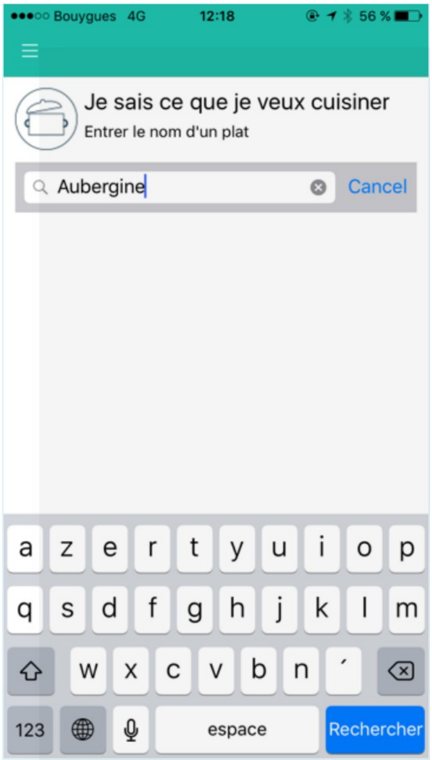

- by ingredient

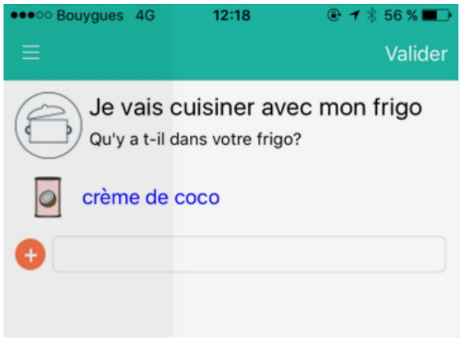

- “context-aware” recipe recommendation feature

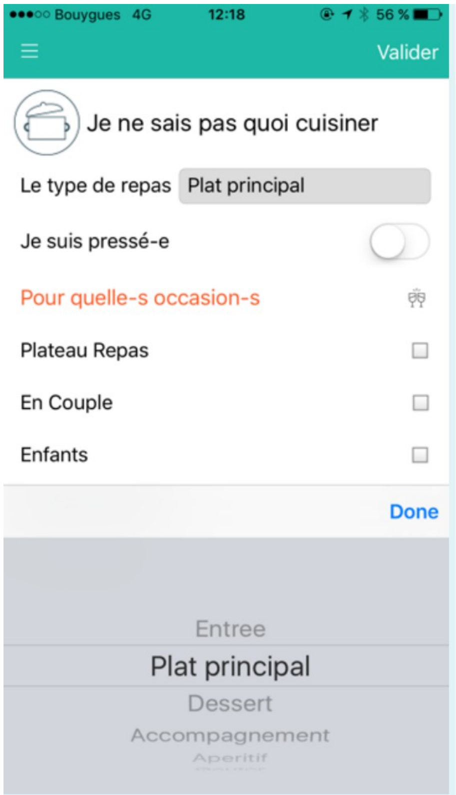

- at random (with the possibility to swipe-out the recipe).

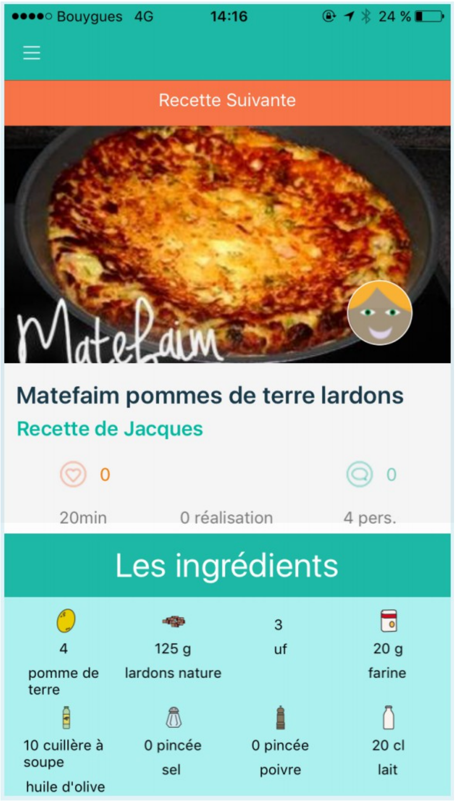

3. Simple nutrition information and cooking tips

Tips were presented through the short and easy to understand notes on (i) the nutritional value of foods or cooking methods (canned fish, steamed cooking); (ii) cooking tips (culinary knowhow, use of cooking utensils…); and (iii) specific information on foods with good nutritional value for price.

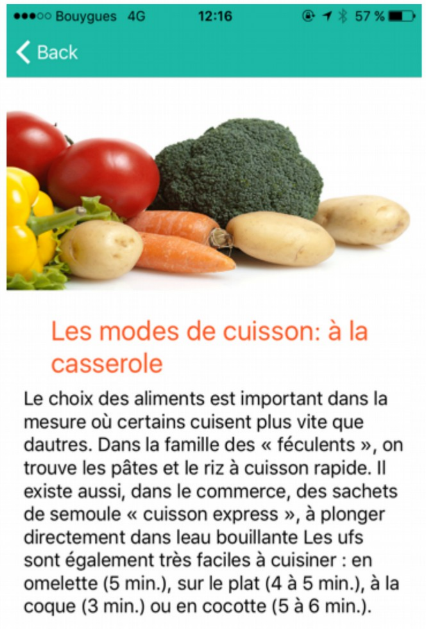

4. Nutrition quizzes

Nutrition quizzes were available; they consisted of short "yes/no" questions followed by a detailed explanatory answer.

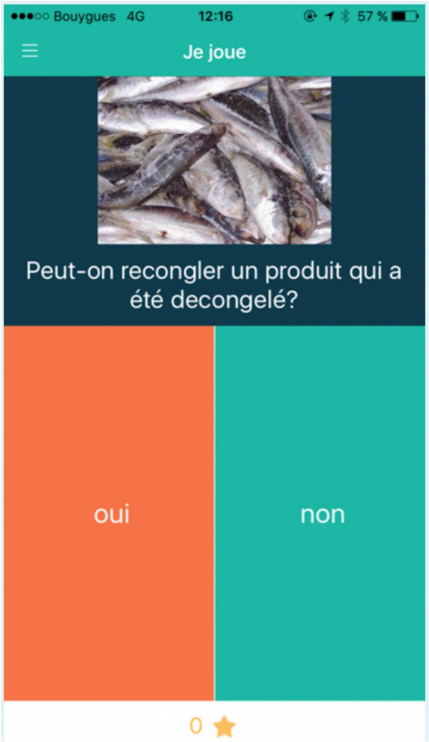

5. Uploading and sharing of recipes

The app allowed users to enter and share recipes, by first adding the general information on the recipe, then listing the ingredients, and finally describing the cooking steps.

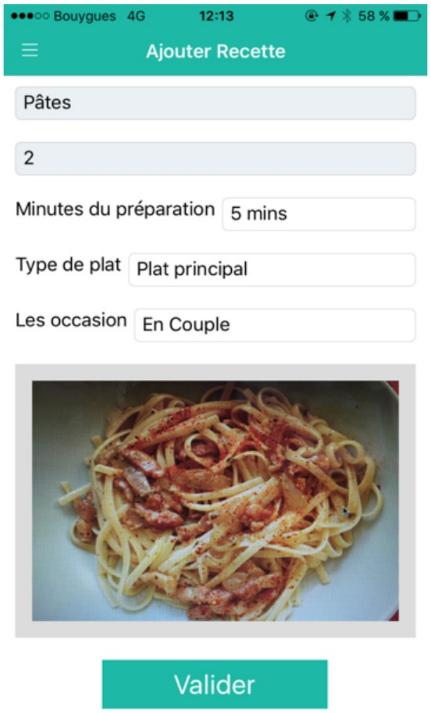

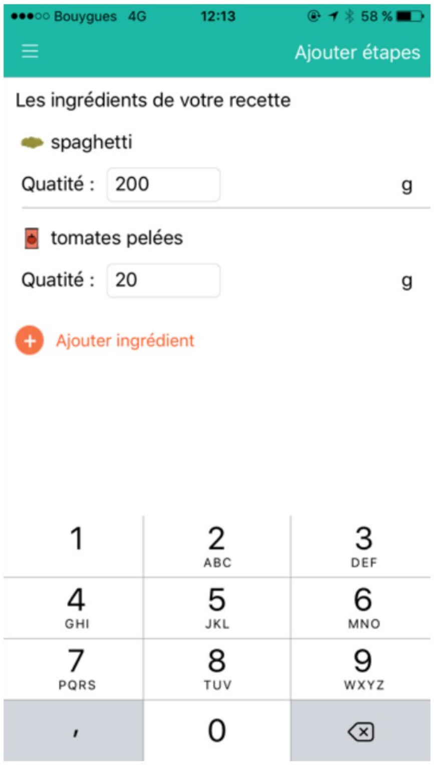

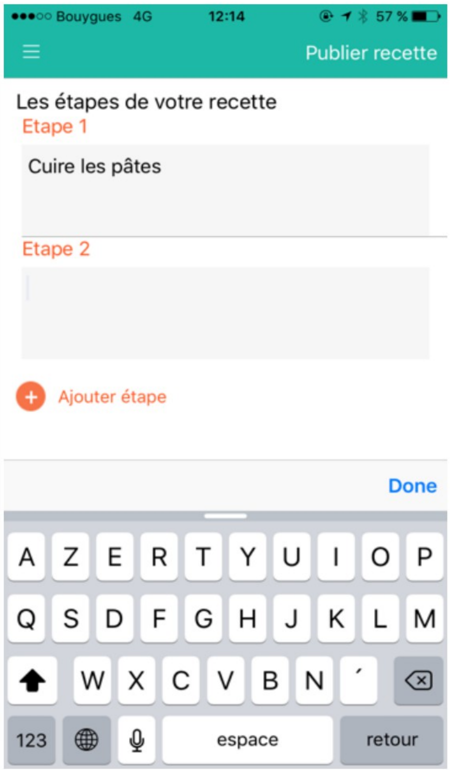

Supplement: Multimedia Appendix 1 [file mhealth_v6i11e11176_app1.pdf]
